# Editorial: Non-Coding RNAs in Neurodevelopmental Disorders

**DOI:** 10.3389/fneur.2017.00629

**Published:** 2017-11-27

**Authors:** Owen M. Rennert, Mark N. Ziats

**Affiliations:** ^1^National Institute of Child Health and Human Development, NIH, Bestheda, MD, United States; ^2^Department of Internal Medicine, University of Michigan, Ann Arbor, MI, United States

**Keywords:** non-coding RNA, RNA, neurodevelopment, neurodevelopmental disorder, neurodevelopmental diseases, epigenesis, genetic, epigenomics

**Editorial on the Research Topic**

**Non-Coding RNAs in Neurodevelopmental Disorders**

The human genome mostly consists of DNA that is transcribed but does not encode for protein. Although originally thought to represent evolutionary “junk,” it has been shown that much of the junk DNA in the human genome is actively transcribed to RNA in a highly regulated, tissue-specific manner (1). Following this insight, non-coding RNAs (ncRNAs) were demonstrated to be fundamental to many intracellular processes, such as targeting transcription factors to their binding sites, initiating chromatin remodeling, blocking transcription or translation of other genes both in cis and trans, and a variety of other functions that are still being uncovered (2). Perhaps not surprisingly, studies quickly followed showing that disruption of ncRNA biogenesis can lead to molecular and cellular defects (3). Recently, ncRNAs have been demonstrated to be abnormal in the brains of patients with common neurodevelopmental disorders and their animal models, such as autism, schizophrenia, and bipolar disorder (4–6). These diseases were previously known to have a significant hereditary component, but their genomic etiology is complex and has remained poorly understood. Emerging research into underlying ncRNA problems in these disorders has the potential to reconcile their known heritability with their genomic and phenotypic heterogeneity, and hopefully unveil novel genomic pathologic mechanisms that can ultimately lead to new molecular therapeutics.

In this Research Topic, a broad array of reviews and new findings in this emerging and critically important area of neurosciences research are presented, underscoring the importance of ncRNAs to our understanding of normal neurodevelopment, and neurodevelopmental and even neurodegenerative disorders. Furthermore, the articles in this Research Topic highlight the varied species of ncRNAs that are likely playing normal and pathologic roles in brain development—from microRNAs (miRNAs) to long non-coding RNAs (lncRNAs) and demonstrate how they interact with chromatin and other transcription machinery to fine tune and process gene expression in the developing brain—and how sometimes this may go awry.

A number of contributions explore the role of ncRNA in the developing brain. Chen et al. demonstrate, in their article “The silencing effect of microRNA miR-17 on p21 maintains the neural progenitor pool in the developing cerebral cortex,” how a specific miRNA is at least partly responsible for regulating the number or progenitor neurons in embryonic mouse cortex. Hect et al. also report original research in their paper “Noncoding RNA in the transcriptional landscape of human neural progenitor cell differentiation,” whereby they use RNA-seq to characterize the ncRNA landscape of human neural progenitor cell lines and discovered through weighted gene co-expression network analysis four modules of RNA transcripts likely to be driving the differentiation process—up to 40% of the RNAs in these modules are non-coding. A timely review of the various miRNA roles in normal neurodevelopmental processes is presented by Davis et al. in “MicroRNAs: not ‘fine-tuners’ but key regulators of neuronal development and function.”

Two other articles demonstrate the importance of ncRNAs not just in early development, but in the continued refinement of the CNS throughout life and as the brain ages. Barry et al., in their original research article “Long non-coding RNA expression during aging in the human subependymal zone,” discovered lncRNAs that appear to be important in coordinating the continued production of neurons throughout adult life from the subependymal zone. In the opinion piece entitled, “Up-regulation of miRNA-146a in progressive, age-related inflammatory neurodegenerative disorders of the human CNS,” Alexandrov et al. review evidence that supports the assertion that miRNA-146a is involved in promoting a pro-inflammatory state that can ultimately lead to neurodegenerative disease such as Alzheimer’s disease and age-related macular degeneration.

Finally, a set of articles explores the role of ncRNAs in neurologic disorders that present across the lifespan. In their review article “Common microRNAs target established ASD genes,” Banerjee-Basu et al. review the broad data implicating miRNAs in autism spectrum disorder pathogenesis and suggest that the heterogeneity of this field necessitates a more systemic evidence-based hierarchy to help guide researchers and clinicians in this ever-evolving field, which subsequently they have gone on to develop (6). Merico et al. present original research on the role of a specific miRNAs affected in 22q11.2 deletion syndrome, which often has schizophrenia as part of the phenotype, in their article “MicroRNA dysregulation, gene networks and risk for schizophrenia in 22q11.2 deletion syndrome.” Finally, Kerschbamer and Biagioli postulate that Huntington’s Disease, which is traditionally considered a neurodegenerative problem, may have features of neurodevelopmental dysregulation that is driven by alterations in ncRNAs, in their opinion piece, “Huntington’s disease as neurodevelopmental disorder: altered chromatin regulation, coding, and non-coding RNA transcription.”

As evidenced by the variety in articles in this Research Topic—spanning neurodevelopment to neurodegeneration, encompassing small and large ncRNAs, and involving both normal development and pathologic processes—the field of ncRNAs in neurodevelopmental disorders is broad and likely to be extremely important to our understanding of normal brain development and brain diseases. The rapid pace of new developments in this field, including the original research presented in this Research Topic, continues to push the boundaries of what is functional RNA, making for exciting opportunities for new discoveries into basic biology as well as new diagnostic and treatment targets.

## Author Contributions

MZ and OR were editors of this research topic and wrote this editorial jointly.

## Conflict of Interest Statement

The authors declare that the research was conducted in the absence of any commercial or financial relationships that could be construed as a potential conflict of interest.
